# Supplementary material for: Environmental characterization of the estuarine zone of the Gulf of Montijo, province of Veraguas, Panama
Source: PLoS One. 2023 Jun 2;18(6):e0283606. doi: 10.1371/journal.pone.0283606 (PMC10237486; doi:10.1371/journal.pone.0283606)
Supplement: Attachment — Submitted filename: Answer to Reviewer comments_PLOS.doc [file pone.0283606.s001.doc]

**Comments Reports for Manuscript PONE-D-21-40756-PLOS**

**Response to Reviewer's Comments:**

**General Comments from the reviewers:**

This manuscript analyzed the vertical and spatial variability of physicochemical parameters along the estuarine zone of the Gulf of Montijo, Province of Veraguas, Panama. There are some serious concerns and points that must be improved before sending the manuscript to the reviewers.

1. **The manuscript fails to identify major achievements in the topic in recent years, major research questions, and future research needs.**

The introduction has been modified to include data regarding Panama and the challenges that the country faces regarding water sustainability and pollution. The following paragraphs have been added to the introduction:

*Most species are characterized by sensitivity to contaminants, relatively broad tolerance for a wide range of salinity, sediment particle sizes and organic matter content, which facilitates tests with various sediment types. Ecological significance (important links in coastal food chains) and wide geographic distribution of some species allows for comparisons throughout regions.(Kraemer et al., 2001; Podlesińska & Dąbrowska, 2019).*

*In Panama, the population has increased by 10 during the last 80 years, especially along coastal areas. With that, commercial human activity has increased; therefore, solid waste has increased, ending up in gulfs. The aforementioned is characteristic of a developing country (Comité de Alto Nivel de Seguridad Hídrica, 2016; Pacheco et al., 2019).*

2. **Unfortunately, the information provided in the introduction and discussion is insufficient to interpret the study correctly.**

The introduction was thoroughly revised to highlight the correlation between population growth, marine-dependent commercial activities (tourism and fishing), and pollution. We focused on measuring physicochemical parameters and the concentrations of heavy metals in the unexposed marine sediment of the western part of the Gulf of Montijo. Likewise, the discussion was enhanced to provide clarity to the document. In the discussion, the results were analyzed and compared to previous studies. Furthermore, the analysis can also be evaluated to develop accurate public policies.

3. **In any case, the Discussion Section must be considerably improved, as it is too general.**

We addressed this challenge in the document.

4. **The manuscript lacks the most recent references; authors are advised to put new concerns from the year 2010-2021 in the manuscript to make it suitable for publication.**

The list of references has been updated. We have included the following references:

1. Bishop, M. J., Mayer-Pinto, M., Airoldi, L., Firth, L. B., Morris, R. L., Loke, L. H. L., Hawkins, S. J., Naylor, L. A., Coleman, R. A., Chee, S. Y., & Dafforn, K. A. (2017). Effects of ocean sprawl on ecological connectivity: impacts and solutions. *Journal of Experimental Marine Biology and Ecology*, *492*, 7–30. <https://doi.org/10.1016/j.jembe.2017.01.021>
2. Collaguazo, Y., Ayala, H., & Machuca, G. (2017). Cuantificación de metales pesados en Anadara tuberculosa (Mollusca:bivalvia) del estero Huaylá de Puerto Bolívar, por espectrofotometría de absorción atómica. Revista Ciencia UNEMI, 10(24), 1–10.
3. Comité de Alto Nivel de Seguridad Hídrica. (2016). Plan Nacional de Seguridad Hídrica 2015-2050. In Gobierno de la República de Panamá. <http://www.conagua.gob.pa/images/documentos/Plan/PRIMER-PLAN-NACIONAL-DE-SEGURIDAD-HIDRICA-DE-LA-REPUBLICA-DE-PANAMA.pdf>
4. Cruz L, N., Alderete C, A., & Laffón L, S. (2013). Accumulation of heavy metals in sediments of mangrove ecosystem in Laguna de Terminos, Campeche, Mexico. Foresta Veracruzana, 15(1), 25–30. http://www.redalyc.org/pdf/497/49728291003.pdf
5. Fuentes-Hernández, M. V., Sanguinetti-Gamboa, O. A., & Rojas De Astudillo, L. L. (2019). Evaluación del riesgo ambiental de metales pesados en los sedimentos superficiales del saco del golfo de Cariaco. Revista Internacional de Contaminacion Ambiental, 35(1), 101–114. https://doi.org/10.20937/RICA.2019.35.01.07
6. González Flores, E., Tornero Campante, M. A., Ángeles Cruz, Y., & Bonilla Y Fernández, N. (2009). Concentración total y especiación de metales pesados en biosólidos de origen urbano. Revista Internacional de Contaminacion Ambiental, 25(1), 15–22.
7. International, C. (2016). Improving mangrove conservation across the Eastern Tropical Pacific Seascape (ETPS) through coordinated regional and national strategy development and implementation. (Issue March).
8. Marqués, M. (2015). Consecuencias de una explotación minera de Zn y Pb ( Mina Troya , Guipúzcoa ).
9. Martínez, G. (2016). Calidad ambiental de los sedimentos recientes del Golfo de Cariaco, Venezuela.
10. McBratney, A. B., & Webster, R. (1983). Optimal interpolation and isarithmic mapping of soil properties: V. Co‐regionalization and multiple sampling strategy. Journal of Soil Science, 34(1), 137–162. https://doi.org/10.1111/j.1365-2389.1983.tb00820.x
11. Morales-Ramírez, Á., Acuña-González, J., Lizano, O., Alfaro, E., & Gómez, E. (2016). Rasgos oceanográficos en el Golfo Dulce, Pacífico de Costa Rica: una revisión para la toma de decisiones en conservación marina. Revista de Biología Tropical, 63(April), 131–160. <https://doi.org/10.15517/rbt.v63i1.23100>
12. Pacheco, B. C. S., Françozo, R. v., Piratelli, C. L., Neyra Belderrain, M. C., & Amaral, A. C. (2019). Value focused performance indicators for graduate programs. Pesquisa Operacional, 39(3), 497–519. https://doi.org/10.1590/0101-7438.2019.039.03.0497
5. **Authors are also suggested to draw major inferences/primary conclusions followed by the secondary conclusions/ recommendations reached through the critical analysis/ investigation of the study.**

The conclusion was revised to highlight the environmental challenges found in the region and its relevance for designing public policies that can bolster sustainability in the region.

The conclusion was revised as follow:

*" Regarding vertical behavior, mainly in the saline structure, it indicates that the estuary is slightly stratified or mixed, which shows that an estuary can present differences at short scales, as in a tidal cycle, and/or at seasonal and temporal scales. The depletion of dissolved oxygen concentrations in anoxia conditions in the inner part and southwest sector of the Gulf is a critical condition for the health of the ecosystem. Since there is an increase in environmental stressors, environmental authorities should be aware of these characteristics and continue with their environmental patrol to strengthen decision-making to protect this aquatic ecosystem. The presence of heavy metals, particularly the strong Cadmium contamination in marine sediments, is critical. It is a fact that environmental stressors due to anthropogenic activity have increased and that there is evidence in the system of bioaccumulation processes in marine biota."* 🡪"*Heavy metal contamination, particularly significant Cadmium contamination in marine sediments, is critical. Environmental stresses caused by human activities have indeed risen and that evidence of bioaccumulation processes in marine biota can be found in the system.*

*The optimal concentrations in the surface layer of the entire OD-pH structure are due to ocean-atmosphere exchange and photosynthetic processes; without leaving aside the dynamics of the sea, which is one of the main forcing factors that affect the distribution of oxygen and pH. Regarding vertical behavior, mainly in the saline structure, we found that the estuary is slightly stratified or mixed, which shows that an estuary can present differences at short scales, as in a tidal cycle, and at seasonal and temporal scales.*

*The depletion of dissolved oxygen concentrations in anoxia conditions in the inner part and southwest sector of the Gulf is a critical condition for the health of the ecosystem. Since there is an increase in environmental stressors, environmental authorities should be aware of these characteristics and continue with their environmental patrol to strengthen decision-making to protect this aquatic ecosystem."*
