# Supplementary material for: Environmental characterization of the estuarine zone of the Gulf of Montijo, province of Veraguas, Panama
Source: PLoS One. 2023 Jun 2;18(6):e0283606. doi: 10.1371/journal.pone.0283606 (PMC10237486; doi:10.1371/journal.pone.0283606)
Supplement: Attachment — Submitted filename: Response to Reviewer-PONE-D-22-11177_24012023.docx [file pone.0283606.s003.docx]

**Response to Reviewer's Comments:**

**Manuscript PONE-D-22-11177-R3**

**Reviewer 1:**

**1. Abstract: "These results show differences with previous studies. In the months of August, October, November
and December...". This brings up the important point that this study was only a snapshot in time (October 17, 2020). The authors describe the need for additional seasonal sampling in the abstract, but this should be highlighted in more detail in the discussion, especially since this is a significant pitfall for this study to accurately characterize environmental conditions for the Gulf of Montijo, which likely vary seasonally.**

Thank you very much for your comments. The discussion was improved accordingly, and the sentence was included in the abstract:

*"The study area requires continuous monitoring representative of seasonality (dry, intermediate, and rainy periods)."*

**2. Results: "the hypoxic dissolved oxygen concentrations (< 1 mg/L) were measured in anoxia condition" is contradictory. In general, dissolved oxygen concentrations <1 mg/L is more accurately described as hypoxic or near anoxic. This was revised in a few instances but not throughout the entire manuscript since the first round of review.**

Thank you very much for the observation. The document was improved accordingly. In all instances, the expression "near anoxia" or "hypoxia" was employed.

**3. Discussion: "These results show differences with previous studies. In the months of August, October, November and December...". This brings up the important point that this study was only a snapshot in time (October 17, 2020). The authors describe the need for additional seasonal sampling in the abstract, but this should be highlighted in more detail in the discussion, especially since this is a significant pitfall for this study to accurately characterize environmental conditions for the Gulf of Montijo, which likely vary seasonally.**

The discussion was modified to include the following paragraphs:

*"The decreases in dissolved oxygen concentrations, mainly in the inner part of the study area, may be due to the crucial contributions of suspended sediments by river discharge. These conditions were observed during the campaign."*

*"Future work focuses on characterizing the estuarine zone during the dry season (summer) and intermediate and rainy seasons to obtain data to analyze the variations that may occur depending on the seasonal period. The most significant changes occur in the inputs of substances that reach the estuary by surface runoff."*

**4. Figures and tables
Figure 3: Why is each environmental parameter labeled as superficial? The figure caption should also mention that these are presumably surface levels of each parameter.**

Thanks for your observations. The Word superficial was removed from Figure 3 and included in the caption. The image was modified as follows:

Figure 3: Horizontal distribution of temperature, salinity, dissolved oxygen, and pH. *Surfer®  (Golden Software, LLC)* under a CC BY license, with permission from *Surfer®  (Golden Software, LLC)*, original copyright *Surfer®  (Golden Software, LLC)*

**Figure 4: Caption includes Spanish.**

Thank you for the observation. The caption was revised accordingly.

**Tables
Table 1: Including Table 1 and a table of coordinates in Figure 1 is redundant.**

Thank you very much for your comment. Figure 1 was modified to include the data in table 1, which was removed from the main text. The image was revised as follows.

Figure 1: Area of Study and Distribution of Stations. Republished from *Surfer®  (Golden Software, LLC)* under a CC BY license, with permission from *Surfer®  (Golden Software, LLC)*, original copyright *Surfer®  (Golden Software, LLC)*

Table 2: Was arsenic (As) measured? If not, then it should be removed from the table. (Not corrected from the first round of review)

We looked for arsenic, but, fortunately, it was not present (or its concentration was extremely low) in the water. Table 1 contains this information.

**Reviewer 2:**

**The authors accepted the suggestions and the manuscript can be accepted for publication. However, the references aren't formatted according to Plos One.**

Thank you very much for your comments.

As for the references, we have used a version of the IEEE format (the references are numbered).
